# Editorial: Exploring the effects of human activities and climate change on soil microorganisms in grasslands

**DOI:** 10.3389/fmicb.2024.1515648

**Published:** 2024-12-12

**Authors:** Shiming Tang, Paul Christiaan Struik, Jie Ren, Chengjie Wang, Ke Jin

**Affiliations:** ^1^Key Laboratory of Model Innovation in Forage Production Efficiency, Ministry of Agriculture and Rural Affairs, Institute of Grassland Research, Chinese Academy of Agricultural Sciences, Hohhot, China; ^2^Centre for Crop Systems Analysis, Department of Plant Sciences, Wageningen University and Research, Wageningen, Netherlands; ^3^College of Ecology and Environment, Inner Mongolia University, Hohhot, China; ^4^College of Grassland Science, Inner Mongolia Agricultural University, Hohhot, China

**Keywords:** grassland, soil microbes, land use change, grazing, climate change

Grasslands, covering over 40% of the Earth's ice-free land area, are critical ecosystems that support biodiversity, sequester carbon, and provide forage for livestock. These ecosystems play a crucial role in the Earth's carbon cycle by storing a substantial amount of organic carbon both above and below ground (Bai and Cotrufo, 2022). However, grasslands are under increasing pressures from human activities such as overgrazing and conversion to cropland, as well as from climate change, leading to significant alterations in the soil-plant-animal system, with strong effects on soil-physical and soil-chemical characteristics and thus on the soil microbiomes of these grasslands (Dijkstra et al., 2023). Soil microbiome is essential for maintaining ecosystem health and function, and its responses to poor management and environmental perturbations can have far-reaching consequences for ecosystem services (Bardgett and van der Putten, 2014). The collection of articles in this Research Topic, “*Exploring the effects of human activities and climate change on soil microorganisms in grasslands*,” provides a comprehensive exploration of these impacts, highlighting the urgency of understanding and mitigating these pressures (Xiang et al.).

The human footprint on grasslands, ranging from overgrazing to conversion for agriculture, is evident in the shifts observed in soil microbial communities. For instance, the introduction of effective microorganisms (EM) as a restoration strategy has shown promise in reviving degraded alpine grasslands, underscoring the potential of microbial inputs to counteract the negative effects of human-induced degradation (Kang et al.). Similarly, the conversion of desert grasslands to agricultural land has profound implications for soil fungal community structures and functions, emphasizing the need for sustainable land management practices to preserve microbial diversity and ecosystem resilience (Xiang et al.).

Climate change, with its associated variations in precipitation and the onset of drought, further complicates the grassland microbiomes. Research within this issue reveals how these climatic fluctuations can disrupt the delicate balance of microbial communities, affecting soil respiration, carbon cycling, and the decomposition of organic matter (Zhuang et al.). The response of these communities to drought stress, particularly the role of functional microbiomes in promoting plant growth under such conditions, is a testament to the adaptability and importance of microbial mediation in grassland ecosystems.

The interactive effects of human activities and climate change on the soil microbiome of grasslands are not solely detrimental; they also present opportunities for microbial-mediated amelioration. The input of litter and the manipulation of precipitation in temperate grasslands can influence microbial communities in ways that may affect ecosystem functions (Gao X. et al.). Moreover, the impact of alternate partial root-zone irrigation on the rhizosphere microbiota of alfalfa plants inoculated with rhizobia highlights the potential for agricultural practices to be tailored to support beneficial microbial populations, thereby improving ecosystem health and productivity (Kang et al.).

Gao H. et al. reported that under mild to moderate drought stress, synthetic microbial communities (SynComs) outperformed single strains in enhancing plant biomass accumulation and inducing the production of resistance-related substances in Pallas' Groundsel (*Neopallasia pectinata*), a dominant forage species in desert steppe. This suggests that SynComs can be leveraged to mitigate drought stress and promote the ecological restoration of these fragile ecosystems. The impact of alternate partial root-zone irrigation (APRI) on the rhizosphere microbiota of alfalfa (*Medicago sativa*) plants inoculated whit rhizobia was investigated by Zou et al.. They found that APRI did not significantly affect the growth of alfalfa in the short term, indicating the potential for agricultural practices to influence beneficial microbial populations.

A deeper understanding of these microbial-mediated processes is crucial for predicting and managing the consequences of environmental change on grassland ecosystems. The hotspots and trends in microbial-mediated grassland ecosystem functions, point to the importance of microorganisms input in degraded alpine grassland (Li et al.). The variations in soil microbial communities among different land use types in the agro-pastoral ecotone of northern China further emphasize the significant role of anthropogenic activities in shaping these ecosystems (Sun et al.).

In conclusion, the research presented in this Research Topic collectively calls for a concerted effort to protect and restore grassland ecosystems in the face of human activities and climate change. The insights gained from these studies are vital for developing strategies that not only mitigate the negative impacts but also harness the potential of soil microorganisms to enhance ecosystem resilience and function. As we continue to navigate the challenges posed by environmental change, the role of soil microbiomes in grassland health must be a central focus of conservation and restoration efforts.
